# Annotations

**Published:** 1897-08-07

**Authors:** 


### Annotations.

### A Good Cry.

Crying is so commonly associated with distress that man's natural instinct is to put a stop to it as soon as possible. We should not forget, however, that it has its uses. Dr. Harry Campbell has recently shown how complex are the phenomena involved in "a good cry." This does not consist merely in the shedding of tears, but includes so general and widespread an action of the muscles that the whole body may be convulsed. In children also a great change takes place during crying in the manner in which the respiration is carried on. Expirations are prolonged sometimes for as much as half a minute, and are interrupted by short inspirations. During expiration the glottis is contracted so that the intrapulmonary pressure rises considerably, and there can be but little doubt that it is the equal distribution of this increased air pressure throughout the whole of the chest, leading to the dilatation of portions of the lung that have become more or less collapsed, that is the explanation of the great benefit which often results from crying in cases of infantile bronchitis and of the large discharge of bronchial mucus which so often follows. Children may become very blue during the paroxysm, but the deep respirations which succeed quickly restore the circulation to a better condition than before in consequence of the larger lung space rendered available. In women the beneficial effect of a good cry is proverbial. In them also this is partly due to the increased depth of respiration and the improvement in the often languid circulation thereby induced, but to a large extent it is the result of the muscular exercise involved, by which the general vascular tension, and especially the blood pressure in the brain, are much reduced. The profuse flow of tears no doubt also acts strongly on the cerebral circulation in still further reducing tension. The sobbing movements, again, have a good influence upon the venous circulation in the abdominal and pelvic viscera, while the exhaustion produced tends to produce sleep, and thus to give the nervous system its best chance of recuperation. We should not, then, too hastily intervene to stop a woman from having out her cry. If we can remove her trouble by all means let us do so; but if the trouble is to remain let her cry herself to sleep. This is far better than soothing draughts.

### Bravery and its Rewards.

The dangers to which medical officers to asylums are exposed in the performance of their daily work are well illustrated by the attack recently made by a lunatic upon Dr. Merson, medical superintendent of the Hull Borough Asylum. While on the cricket field Dr. Merson received a violent blow on the back of his head from one of the patients, the blow being struck with a cricket bat. Dr. Merson was rendered unconscious for several hours, and remained for some days in such a condition as to cause great anxiety. We are glad to hear, however, that he is now progressing favourably. Great as are the advantages of the humane methods of treating the insane now generally adopted in our modern asylums, the public ought to recognise that in the comparative freedom from obvious restraint which insane patients now so commonly enjoy, there are elements of danger to those who have to deal with them. When we consider the question of pension for asylum officials we ought always to bear in mind that, monotonous as their life may seem, and in fact may actually be, it is a life passed in the midst of ever present danger and under a continued sense of strain. The highest honours, and in some sense the highest rewards, are given by a grateful nation to displays of bravery and self-abnegation which, after all, are often due to sudden impulse, to inborn indifference to danger, almost, one might say, to a reversion in times of great excitement to those animal instincts of pugnacity and unreasoning courage which we share with the terrier, the bull dog, and other noble animals. The steady and persistent bravery which leads men of science to walk with unflinching step through a whole lifetime of peril, which enables them to face typhus and diphtheria, cholera and plague without fear or hesitation, and to listen quietly and critically to the fantastic conceits of lunacy, while conscious all the time that a fatal blow may be struck or wound inflicted at any moment, this is what the nation does not recognise and does not appear to understand; for it is bravery of this kind that the nation rewards, not with open-handed pensions, but with petty hagglings over the mean details of those superannuation Bills by which the servants of the public are now made to provide their own rewards.

### The Harrogate Royal Baths.

The Corporation of Harrogate is to be congratulated on the completion of the Royal Baths, which have been in course of erection for the past two or three years. It is a matter of much more than mere local importance that the medicinal baths and waters of England should not be allowed to run to waste. Few who have not visited foreign bathing establishments have any idea of the enormous amount of English money which is annually spent in searching for health abroad, which might just as well be, and possibly would be, spent at home if English bathing-places provided the same facilities and the same attractions as are to be found in foreign spas. By dint of organisation and a little expenditure on music, paths, and promenades, many a Continental watering-place, the therapeutic efficacy of whose waters is absolutely problematical, keeps itself in good repute among searchers after health; and it may almost be called a national misfortune that our English waters, some of which, like Harrogate, possess unrivalled medicinal properties, should not be more patronised than they are. The difficulty is largely one of organisation. The healthy tripper and the health-seeking invalid do not harmonise together. It is hardly too much to say that they are absolutely incompatible, and it is to be hoped that those who are responsible for the management of our health resorts will sooner or later see the folly of catering for trippers and occasional visitors. Let such people go where they can enjoy themselves, but let them be kept at arm's length wherever nature gives facilities for the cure of disease. Not that such places need be dull, but what is pleasure to the half-day tripper is absolute torture to those undergoing a three weeks' "cure." It is with extreme satisfaction then that we find that the Baths Committee of the Harrogate Corporation have sunk £100,000 in the new establishment. Such an expenditure pins them down to a policy—one which shall encourage those who come for a "cure" and shall discourage those who come for a "lark"—for their interest can only come out of the pockets of those who seek for health. That £100,000 is a hostage to scientific balneology.

